# Loss of TDP-43 Drives Innate Immune Activation Through Relish in *Drosophila*

**DOI:** 10.3390/ijms27125359

**Published:** 2026-06-13

**Authors:** Giulia Romano, Raffaella Klima, Fabian Feiguin

**Affiliations:** 1 International Centre for Genetic Engineering and Biotechnology, Padriciano 99, 34149 Trieste, Italy; 2 Neuronal Aging and Neurodegeneration Laboratory, Department of Life and Environmental Sciences, University of Cagliari, 09042 Monserrato, Italy

**Keywords:** TDP-43, ALS, FTD, neurodegeneration, NFkB, Relish, neuroinflammation, microarray, *Drosophila*

## Abstract

Inflammatory and immune alterations are increasingly recognized as components of ALS pathology, yet whether they arise as a direct consequence of TDP-43 dysfunction or as a downstream response to neurodegeneration remains unresolved. To address this question, we profiled adult head transcriptomes of *Drosophila* lacking TBPH, the fly homolog of TDP-43, and identified marked overactivation of the conserved Toll/Imd/NF-κB (Relish) innate immune pathway, including increased expression of antimicrobial effector genes and inflammatory genes. We further found that TDP-43/TBPH regulates the NF-κB homolog Relish by associating with its mRNA and that its loss permits Relish-dependent immune overactivation. Genetic reduction in Relish in TDP-43-deficient flies suppressed inflammatory signaling and ameliorated neurological defects *in vivo*, indicating that immune dysregulation contributes to TDP-43 loss-associated phenotypes.

## 1. Introduction

Amyotrophic lateral sclerosis (ALS) is a progressive and fatal neurodegenerative disorder characterized by the degeneration of upper and lower motor neurons, leading to muscle weakness, paralysis, and ultimately respiratory failure [1,2]. Despite substantial advances in defining the genetic and pathological landscape of ALS, the molecular mechanisms that drive neuronal vulnerability and disease progression remain incompletely understood. One of the most prominent pathological features of ALS is the dysfunction of the RNA-binding protein TDP-43, which is mislocalized from the nucleus and accumulates in cytoplasmic inclusions in the vast majority of ALS cases, as well as in many cases of frontotemporal degeneration (FTD) [3,4]. Pathogenic TDP-43 mutations identified in both familial and sporadic ALS have further been established as having causal relevance to this disease [5], placing TDP-43 at the center of current models of pathogenesis.

TDP-43 is a predominantly nuclear RNA-binding protein with affinity for UG-rich sequences, broadly involved in transcriptional regulation, pre-mRNA splicing, RNA transport, and mRNA stability [6,7]. A disease-relevant function of particular importance is the repression of cryptic exon inclusion: nuclear TDP-43 loss of function induces aberrant splicing and depletion of transcripts essential for neuronal integrity, including *UNC13A* and *STMN2*, whose disruption compromises synaptic function and axonal maintenance respectively [8,9,10]. Consequently, TDP-43 dysfunction is expected to disrupt broad gene regulatory networks essential for neuronal homeostasis; however, the downstream cellular pathways that translate this dysfunction into neurodegeneration are still not fully resolved.

Among the pathways increasingly implicated in ALS, neuroinflammation has emerged as a major component of disease pathology. Immune activation, glial reactivity, and inflammatory signaling have been documented in patient tissue and in multiple experimental models, suggesting that non-cell-autonomous mechanisms participate in disease progression [11,12]. Multiple mechanisms connect TDP-43 pathology to immune signaling, including cytoplasmic TDP-43-driven mitochondrial DNA release and cGAS–STING activation [13], PKR-dependent inflammatory signaling via dsRNA accumulation upon TDP-43 depletion [14], and microglial NF-κB and NLRP3 inflammasome engagement by aggregated or extracellular TDP-43 [15]. Consistently, glial NF-κB signaling contributes to ALS progression [16], and chronic NF-κB activation is a shared feature across multiple neurodegenerative disorders, including Alzheimer’s disease [17]. Yet an important conceptual question remains unanswered: does inflammatory activation simply reflect a downstream response to neurodegeneration, or can it be engaged more directly by primary defects in TDP-43 function? Resolving this issue bears directly on how ALS pathogenesis is understood and on whether inflammatory pathways represent accessible therapeutic targets upstream of neuronal damage.

The study of this problem benefits from model systems in which conserved molecular pathways can be interrogated with precision in vivo. *Drosophila melanogaster* provides a powerful framework for this purpose, combining strong genetic tractability with highly conserved regulatory programs relevant to neurodegeneration. The fly ortholog *tbph* is functionally conserved, and its loss produces motor and neuromuscular defects consistent with endogenous loss of function; age-dependent reductions in TBPH/TDP-43 precede locomotor decline [18], and sequestration of endogenous TBPH into aggregates induces locomotor impairment and reduced lifespan [19]. Innate immune signaling in flies is coordinated by the Toll and Imd pathways, which converge on NF-κB-related transcriptional programs controlling inflammatory and antimicrobial gene expression [20,21], and accumulating evidence indicates that these pathways also influence tissue homeostasis, aging, and neuronal integrity. Previous studies demonstrated that neuronal TDP-43 overexpression induces innate immune gene upregulation and that suppression of Toll/Dif or Imd/Relish signaling mitigates toxicity [22] and that MEK/ERK inhibition reduces antimicrobial peptide induction and improves phenotypes [23]. However, these studies primarily relied on overexpression models, leaving open whether endogenous *tbph* loss is sufficient to drive a coherent, rescue-sensitive immune transcriptional program.

To address this question, we combined transcriptomic profiling of *tbph* loss-of-function mutants with conditional, neuron-restricted TBPH re-expression to distinguish primary, reversible consequences of TBPH dysfunction from secondary effects and used genetic and biochemical assays to define the underlying regulatory mechanism.

## 2. Results

We previously generated and extensively characterized a *Drosophila* model for ALS and FTD by creating two loss-of-function alleles of *tbph*, the highly conserved *Drosophila* homolog of TDP-43 [24]. The null mutants recapitulate the major features of neurodegenerative processes, and expression of the human or endogenous protein in the knockout fly rescues most pathological phenotypes, including locomotor defects, reduced lifespan, synaptic abnormalities, and central brain alterations [19,24,25,26,27,28,29,30,31,32,33].

**Figure 1:**
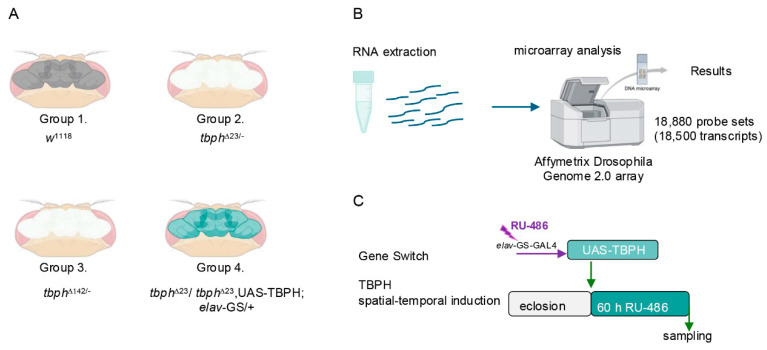
Experimental design for microarray analysis in the Drosophila TBPH mutant model. (**A**) Schematic representation of the four experimental groups used for transcriptomic profiling of adult head tissues. Group 1, wild-type control (*w*^1118^); group 2, *tbph*^Δ23^ null mutant; group 3, *tbph*^Δ142^ null mutant; group 4, conditional pan-neuronal rescue (*tbph*^Δ23^, UAS-TBPH/*tbph*^Δ23^; *elav*-GS-GAL4). (**B**) Workflow of the transcriptomic analysis. Total RNA was extracted from adult head tissues and subjected to microarray analysis using the Affymetrix *Drosophila* Genome 2.0 Array, comprising 18,880 probe sets covering approximately 18,500 transcripts. (**C**) Conditional induction strategy for pan-neuronal TBPH re-expression. In *tbph*^Δ23^ mutants carrying *elav*-GS-GAL4 and UAS-TBPH, TBPH expression was induced by administration of RU-486 immediately after eclosion and maintained for 60 h prior to sampling, enabling temporally controlled and neuron-specific rescue.

To define the transcriptional consequences of *tbph* loss, we performed microarray analysis on head tissues from four groups: wild-type controls (*w*^1118^), two independent *tbph* mutant alleles (*tbph*^Δ23^ and *tbph*^Δ142^), and a conditional neuronal rescue group in which *tbph* mutant carried a UAS-TBPH transgene driven pan-neuronally by *elav*-GS-GAL4 (Figure 1A–C) [34,35]. The GeneSwitch system temporally controlled re-expression of TBPH exclusively in adult neurons, bypassing any contribution of developmental TBPH activity: induction was initiated immediately after eclosion by feeding 5 mM RU-486 and maintained for 60 h before sampling. This time window has been previously shown to be sufficient to restore TBPH protein levels and to significantly rescue locomotor performance in climbing assays [32], supporting its adequacy to capture functionally meaningful transcriptional reversion. This design therefore tests the sufficiency of adult neuronal TBPH to restore the transcriptome altered by constitutive *tbph* loss.

RNA from three biological replicates per group was analyzed using the Affymetrix Drosophila Genome 2.0 Array (~18,500 transcripts). Quality control confirmed clear sample segregation by PCA, low technical variability (RLE/NUSE), and consistent clustering of the two *tbph* alleles (Supplementary Figure S1).

### 2.1. Differential Gene Expression Analysis Identifies a Common Mutant Core and a Transcriptional Rescue Signature

**Figure 2:**
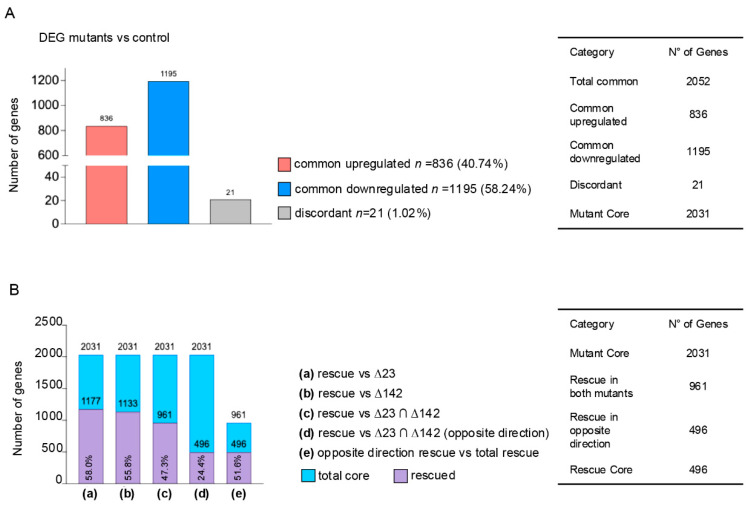
Comparative differential gene expression analysis of DEG mutants relative to control. (**A**) Overlap of differentially expressed genes (DEGs) between ∆23 (*tbph*^∆23/∆23^) vs. control (*w*^1118^) and ∆142 (*tbph*^∆142/∆142^) vs. control (*w*^1118^). Bar plot shows the number of genes commonly regulated in both mutants relative to control: 836 commonly upregulated genes, 1195 commonly downregulated genes, and 21 discordant genes exhibiting opposite directions of regulation between mutants. The summary table (right) reports the numerical distribution of genes across categories. (**B**) Rescue analysis of shared DEGs. Stacked bar plots display the total number of mutant commonly regulated genes (*n* = 2031 per comparison, excluding discordant genes) and the subset rescued under different genetic conditions: (a) rescue vs. Δ23, (b) rescue vs. Δ142, (c) rescue vs. Δ23 ∩ Δ142 (both mutants), (d) rescue vs. Δ23 ∩ Δ142 with opposite direction in rescue compared to mutants, and (e) opposite direction rescue vs. total rescue. Cyan bars represent the total shared DEG core, and purple bars indicate the number of genes rescued in each condition (percentage reported for each). The summary table (right) reports the numerical distribution of genes across categories.

Differential expression analysis was performed using limma after RMA normalization, with probe sets collapsed to gene-level summaries. Comparing each mutant allele to wild-type controls defined a common mutant core of 2031 genes significantly deregulated in both backgrounds: 836 commonly upregulated and 1195 commonly downregulated, with only 21 discordantly regulated genes, confirming strong transcriptional concordance between independent *tbph* alleles (Figure 2A). Unsupervised clustering of the top 300 most highly expressed genes clearly separated mutant from control animals (Supplementary Figure S2).

Comparing the neuronal rescue condition to each mutant background defined a rescue core of 496 genes whose expression was significantly reversed toward control values in both rescue-versus mutant comparisons (Figure 2B). This nested subset (~24% of the mutant core) isolates the transcriptional changes that are causally dependent on TBPH function and reversible by neuronal re-expression within the 60 h induction window and therefore distinguishes primary, TBPH-responsive alterations from secondary, compensatory, or developmentally fixed changes that persist despite restoration. Complete gene lists are reported in Supplementary Table S1.

### 2.2. Functional Characterization of the Common Mutant Transcriptional Core

**Figure 3:**
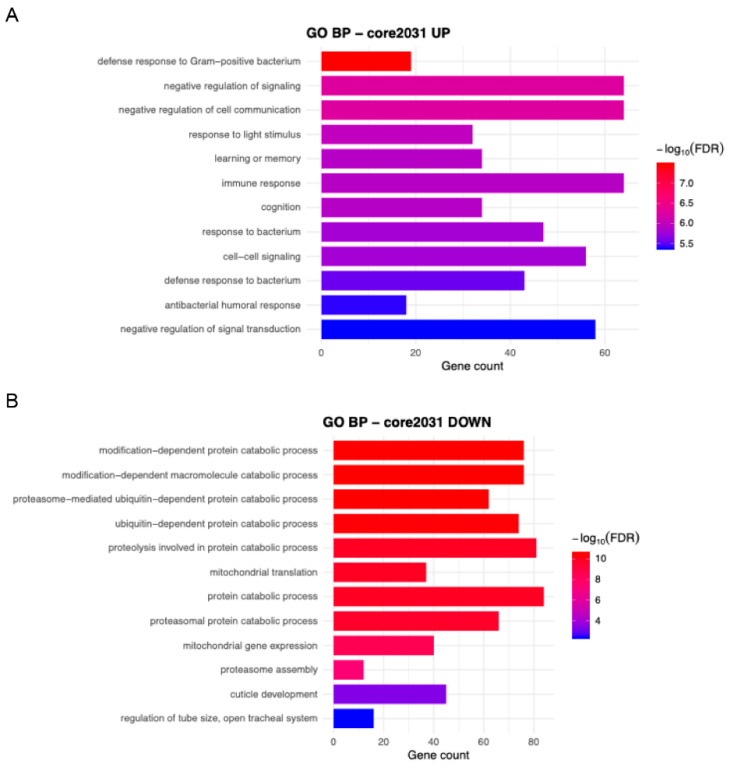
Gene Ontology (GO) enrichment analysis of concordantly regulated genes in *tbph*^Δ23^ and *tbph*^Δ142^ mutants relative to *w*^1118^. (**A**) GO BP terms enriched among genes commonly upregulated in both *tbph* mutants. Enriched categories are dominated by immune and stress-associated processes, including defense response to Gram-positive bacterium, immune response, antibacterial humoral response, response to bacterium, and regulation of cell communication and signaling. (**B**) GO Biological Process (BP) terms enriched among genes commonly downregulated in both *tbph* mutant backgrounds. Enriched categories are predominantly related to proteostasis and metabolic functions, including ubiquitin-dependent protein catabolic processes, proteasome-mediated protein catabolism, proteasome assembly, protein catabolic processes, mitochondrial translation, and proteolysis. In both panels, bar length indicates gene count, and color denotes −log10(FDR-adjusted *p*-value.

GO enrichment analysis of the 2031 concordantly deregulated genes revealed two opposing transcriptional programs. Upregulated genes were enriched for two parallel programs: immune and defense response (defense response to Gram-positive bacterium, immune response, antibacterial humoral response, and defense response to bacterium) and signaling/communication regulation (negative regulation of signaling, of cell communication, and of signal transduction), with additional terms related to neuronal cognitive functions (learning or memory; cognition) (Figure 3A), with Cellular Component enrichment pointing to neuronal and synaptic compartments such as neuron projections, synapse, and presynapse (Supplementary Figure S3B,D). Downregulated genes were predominantly enriched for proteostasis-related processes, including ubiquitin-dependent protein catabolism, proteasome-mediated degradation, and mitochondrial translation, with Molecular Function terms including structural constituents of the ribosome and peptidase activities (Figure 3B and Figure S3A,C).

Together, these data define a transcriptional program characterized by activation of immune and signaling pathways alongside coordinated downregulation of proteostasis and ribosomal systems, consistent with the broad impact of TBPH loss on neuronal homeostasis.

### 2.3. Rescue Selectively Normalizes Toll/Imd–Relish Signaling

**Figure 4:**
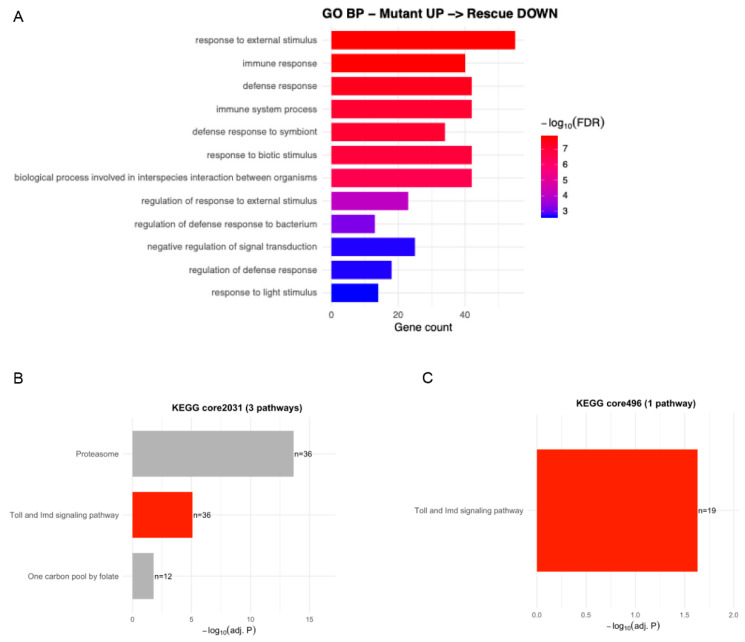
GO and KEGG enrichment identify innate immune signaling as the selectively rescued module. (**A**) GO Biological Process (BP) enrichment analysis of genes oppositely regulated in the rescue background relative to *tbph* mutants. Enriched categories are dominated by immune- and defense-related processes, including immune response, defense response, immune system process, response to external stimulus, and regulation of defense response. Bar length indicates gene count, and color denotes −log10(FDR-adjusted *p*-value). (**B**) KEGG pathway enrichment analysis of the shared 2031-gene mutant core. Three pathways are significantly enriched: proteasome, Toll and Imd signaling pathway, and one carbon pool by folate. Bar length indicates −log10(adjusted *p*-value), and numbers denote gene counts per pathway. (**C**) KEGG pathway enrichment analysis of the 496 rescue-reversed genes. Toll and Imd signaling pathway is the only significantly enriched pathway in this subset.

We next asked which components of the mutant transcriptional program are responsive to neuronal TBPH re-expression. Functional analysis of the 496 rescue-sensitive genes revealed a highly coherent immune signature: GO enrichment was dominated by immune and defense-related Biological Processes, including immune response, defense response, and regulation of defense response (Figure 4A). No significant enrichment was detected in the opposite direction (genes downregulated in mutants and restored upward by rescue), indicating that rescue acts primarily by suppressing aberrantly activated pathways rather than restoring repressed ones.

KEGG enrichment analysis further refined these findings: while the full 2031-gene mutant core showed enrichment for multiple pathways including proteasome, one carbon pool by folate, and Toll/Imd signaling (Figure 4B), the 496-gene rescue subset converged on a single significantly enriched pathway—Toll and Imd signaling (Figure 4C). Expression mapping onto the KEGG pathway architecture confirmed coordinated upregulation across upstream receptors, intracellular mediators, and downstream AMP targets in mutants, with these same components returning toward control levels upon rescue (Supplementary Figure S4A,B), most consistently along the Imd–Relish branch (Supplementary Figure S4C). These data indicate that innate immune signaling represents the most selectively reversible component of the mutant transcriptional landscape. 

### 2.4. relish and Downstream AMPs Are Induced in tbph Mutants and Reverted upon TBPH Re-Expression

**Figure 5:**
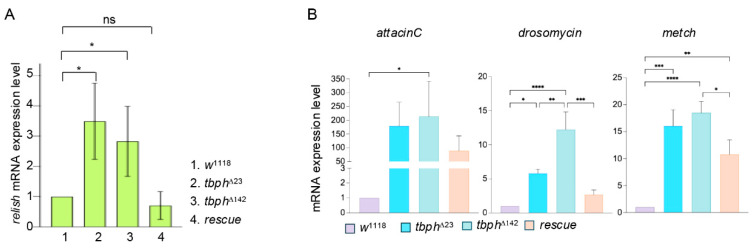
qPCR validation of Relish pathway activation and rescue in adult heads. (**A**) Quantitative PCR (qPCR) analysis of *relish* transcript levels in adult heads from *w*^1118^, *tbph*^Δ23^, *tbph*^Δ142^, and rescue flies (*tbph*^Δ23^, UAS-TBPH/*tbph*^Δ23^; *elav*-GS-GAL4). Expression levels were normalized to *rpl32*. Data represent mean ± SEM (*n* = 3 biological replicates). (**B**) qPCR analysis of canonical downstream targets of Relish signaling in adult heads: *Attacin C*, *Drosomycin*, and *Metchnikowin*. Expression levels were normalized to *rpl32.* Data represent mean ± SEM (*n* = 3 biological replicates). Statistical significance was assessed by one-way ANOVA (ns = not significant, * *p* < 0.05, ** *p* < 0.01, *** *p* < 0.001, **** *p* < 0.0001).

To validate the transcriptional alterations identified by microarray, we performed qRT-PCR on selected components of the Relish-dependent innate immune pathway in adult heads. Consistent with the microarray data, *relish* mRNA levels were significantly elevated in both *tbph*^Δ23^ and *tbph*^Δ142^ mutants compared to *w*^1118^ controls (Figure 5A). Expression of the *Drosophila* ortholog of human TDP-43 (60 h induction) reduced *relish* transcript levels toward control values, demonstrating that the observed activation is functionally reversible.

Canonical AMP genes downstream of Relish—Attacin C, Drosomycin, and Metchnikowin—were strongly upregulated in both mutant lines, consistent with activation of the Imd/Relish axis (Figure 5B). Upon rescue, AMP expression showed a consistent downward trend across all tested targets, supporting coordinated attenuation of the pathway.

Together, these results independently validate the microarray findings and confirm that TBPH loss induces a Relish-dependent innate immune transcriptional program in adult heads, the activation of which is dampened by TBPH re-expression.

### 2.5. Genetic and Biochemical Evidence Linking TBPH Dysfunction to Relish-Dependent Immune Activation and Locomotor Impairment

**Figure 6:**
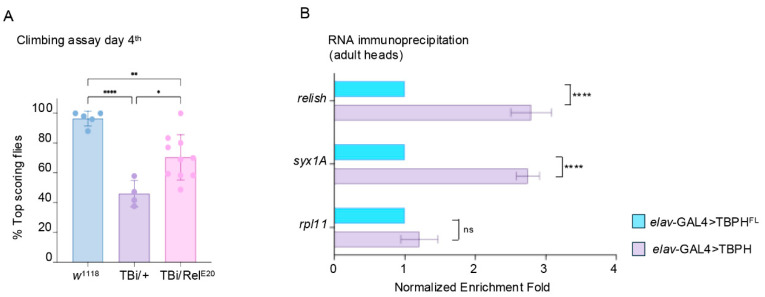
Genetic and biochemical evidence linking TBPH to Relish-dependent immune activation. (**A**) Negative geotaxis performance was assessed at 4 days of age. TBi/+ animals (*tbph*^Δ23^, *elav*-GAL4/+; UAS-Dicer2,UAS-TBPH-IR/+) show a significant reduction in climbing ability compared to *w*^1118^ controls, whereas TBi/Rel^E20^ animals (*tbph*^Δ23^, *elav*-GAL4/Rel^E20^; UAS-Dicer2, UAS-TBPH-IR/+) display partial rescue of motor performance. Data represent >100 flies per group. (**B**) RNA-immunoprecipitation (RIP) from adult heads. Normalized enrichment fold from RIP assays assessing interaction of neuronal TBPH protein with *relish* and *syntaxin 1A* (*syx1A*) mRNAs. *elav*-GAL4>TBPH (*elav*-GAL4/UAS-TBPH) shows strong and significant enrichment of both *relish* and *syx1A* compared to *elav*-GAL4>TBPH^FL^ (*elav*-GAL4/+;UAS-TBPH^FL^) control, whereas *rpl11* serves as a non-significant negative control. Data represent mean ± SEM (*n* = 3 biological replicates). Statistical significance is indicated (ns = not significant, * *p* < 0.05, ** *p* < 0.01, **** p < 0.0001).

To assess the functional contribution of Relish-dependent immune activation to the behavioral phenotype associated with TBPH dysfunction, we performed genetic interaction experiments using a hypomorphic *tbph* allele (*tbph*^Δ23^, *elav*-GAL4/+; UAS-Dcr2, UAS-TBPH RNAi/+) [30]. Because *tbph* null mutants show severely compromised locomotion that saturates the climbing assay and masks modifier effects, this hypomorphic background preserves a measurable motor window in which climbing ability was assessed at 4 days of age as an index of motor function. Hypomorphic flies displayed significant impairment in negative geotaxis compared to *w*^1118^ controls (Figure 6A), and genetic reduction in Relish (*relish*^E20^ heterozygous background) partially rescued locomotor performance, establishing a causal link between *tbph* dysfunction and Relish-dependent signaling in vivo. Although relE20/+ animals were not independently assayed in the present study, this configuration has been reported not to affect locomotor performance in otherwise wild-type flies [36,37], making haploinsufficiency of relish an unlikely confounding factor.

To investigate whether this interaction reflects a direct molecular relationship, we performed RNA immunoprecipitation on adult head extracts. TBPH immunoprecipitates showed significant enrichment of *relish* transcripts compared to the RNA-binding–deficient TBPH^F/L^ mutant control (Figure 6B and Figure S5), while a negative control transcript (*rpl11*) showed no enrichment, confirming specificity. Enrichment of *syntaxin 1A* (*syx1A*), a known TBPH target [32], was used to validate the assay.

Together, these data support a model in which TBPH associates with *relish* mRNA, restraining its expression and downstream immune activation and consequent locomotor deficits.

## 3. Discussion

Using a *tbph* loss-of-function model, we identify aberrant Imd–Relish (NF-κB) activation as the dominant rescue-sensitive transcriptional consequence of *tbph* deficiency, supported by pathway reversal upon neuronal TBPH re-expression and by genetic and biochemical validation. These findings indicate that immune dysregulation is not merely an associated feature of TDP-43 pathology but part of the molecular cascade through which neuronal dysfunction is produced.

### 3.1. TBPH Restrains Relish-Dependent Innate Immune Signaling

Across independent *tbph* alleles, Imd/Relish signaling and Relish-dependent AMPs are induced, and this module is selectively attenuated by neuronal TDP-43 homologue re-expression. Functionally, Relish reduction ameliorates locomotor impairment, and TBPH associates with *relish* mRNA, supporting post-transcriptional regulation. While Relish protein cleavage and nuclear translocation were not directly assessed—and this represents a direction for future work—coordinated induction of *relish* and AMP targets across two independent alleles, their attenuation upon neuronal TBPH re-expression, and locomotor rescue by Relish reduction provide convergent functional evidence for Relish-dependent transcriptional activation. This interaction is consistent with the broader view of TDP-43 as a multifaceted post-transcriptional regulator beyond cryptic splicing control.

Beyond the immune module, the broader mutant transcriptional signature encompassed coordinated downregulation of proteostasis and synaptic programs, consistent with established roles of TDP-43 in neuronal maintenance across species and model systems [3,4,9,24,38,39,40,41,42,43]. The selective rescue of the immune module, however, positions Imd–Relish activation as the most functionally reversible and mechanistically proximal consequence of TBPH deficiency. Several non-mutually exclusive mechanisms likely contribute to this selectivity. First, *relish* mRNA is associated with TBPH (Figure 6B), whereas the proteostasis transcripts are more plausibly repressed as a secondary consequence of proteotoxic stress arising during the LOF state: accumulation of misfolded substrates and depletion of the free ubiquitin pool engage integrated stress and unfolded-protein responses that themselves suppress biogenesis programs, and this substrate backlog can persist beyond 60 h, sustaining repression of the proteostasis module independently of TBPH availability. Second, the rescue paradigm is by design neuron-restricted (*elav*-GS-GAL4), whereas the transcriptomic readout integrates contributions from glia, fat body, and hemocyte populations that remain TBPH-deficient. Together, these considerations frame Imd–Relish activation as the most directly reversible output of TBPH loss and motivate a closer examination of how TDP-43 intersects with NF-κB signaling in neurodegeneration.

Previous studies have linked TDP-43 dysfunction to immune overactivation: neuronal TDP-43 overexpression induces innate immune genes, and suppression of Toll/Dif or Imd/Relish signaling mitigates toxicity [22]. Inhibition of MEK/ERK signaling reduces AMP induction and improves phenotypes in TDP-43 models [23], positioning immune activation downstream of stress kinase pathways. Our loss-of-function data complement these findings by identifying Relish-dependent immunity as a primary and reversible output of TBPH deficiency. Three important considerations deserve mention. First, the constitutive *tbph* null is absent throughout development, whereas TDP-43 dysfunction in ALS is adult-onset; the ~76% of the mutant core not reversed by rescue likely includes developmental and compensatory changes, and adult-stage restricted knockdown would more directly model disease context. Second, the whole-head transcriptomic readout integrates contributions from multiple cell types while the rescue is neuron-specific, so the rescue-sensitive signature captures the neuronal component but does not exclude glial or humoral contributions. Third, the microarray rescue experiment lacked driver-only and UAS-only RU486 controls, and a formal contribution of GeneSwitch activity to the rescue signature cannot be excluded. Furthermore, although the GAL4/UAS system provides a controlled means to drive TBPH re-expression, it does not fully recapitulate the chromatin and regulatory context of endogenous promoters, and conclusions should be interpreted within the limits of this heterologous expression system. Nevertheless, the convergence of qPCR, genetic rescue by Relish heterozygosity, and RIP provides independent support for the biological specificity of the finding.

### 3.2. TDP-43, NF-κB, and Innate Immunity in Neurodegeneration

In mammalian systems, TDP-43 pathology engages innate immunity through multiple converging mechanisms. Cytoplasmic TDP-43 promotes mitochondrial DNA release and cGAS–STING activation [13], TDP-43 depletion induces PKR-dependent immune activation via dsRNA accumulation [14], and extracellular or aggregated TDP-43 activates microglial NF-κB and NLRP3 inflammasome pathways [15]. Our data add an upstream regulatory dimension by identifying *relish* mRNA as a TBPH-associated transcript and demonstrating genetic suppression of behavioral deficits via Relish reduction. In ALS, NF-κB signaling contributes to motor neuron toxicity through glial activation [16], NF-κB pathway activation is genetically and environmentally linked to ALS risk [44], and neuroinflammatory mechanisms drive disease progression [12]. Positioning TDP-43 upstream of NF-κB regulation suggests that immune dysregulation may be embedded within the primary molecular consequences of TDP-43 loss rather than representing a downstream stress response. Chronic NF-κB activation and sterile innate immune pathways, including nucleic-acid sensing and inflammasome activation, are increasingly recognized as shared drivers across ALS and related neurodegenerative conditions [45], further supporting the relevance of this regulatory axis. Although the immune signature identified here likely reflects integrated multi-tissue responses given the whole-head transcriptomic approach, the attenuation of immune gene induction by neuronal TBPH re-expression, together with evidence that glial TDP-43 expression independently induces inflammatory phenotypes and neurodegeneration in *Drosophila* [46,47], supports a model in which neuronal TBPH loss is sufficient to drive broader inflammatory activation.

Collectively, these findings support a conserved model in which TDP-43/TBPH maintains innate immune equilibrium by restraining NF-κB/Relish signaling at the RNA level. Loss of this control permits chronic immune activation contributing to neurodegenerative phenotypes, whereas re-expression restores immune balance. Therapeutically, this axis appears actionable: inhibition of NF-κB-inducing kinase (NIK) improves motor performance in ALS models [48], neuron-specific NF-κB inhibition mitigates behavioral and pathological alterations in TDP-43 contexts [49], and pharmacological NF-κB inhibition ameliorates motor and inflammatory phenotypes in ALS-CSF paradigms [50], reinforcing NF-κB modulation as a disease-modifying axis.

In summary, we identify Relish-dependent innate immune activation as a central, reversible, and mechanistically grounded component of TBPH-mediated neurodegeneration, bridging *Drosophila* genetics with conserved inflammatory pathways implicated in TDP-43 proteinopathies.

## 4. Materials and Methods

### 4.1. Microarray Processing and Normalization

Raw Affymetrix CEL files (Drosophila Genome 2.0 Array; platform Drosophila_2) were imported into R and processed using Bioconductor [51]. Background correction, quantile normalization, and probe-level summarization were performed using the Robust Multi-array Average (RMA) algorithm [52], generating log2-transformed expression values stored in an ExpressionSet object. Quality control was assessed by inspection of expression distributions and replicate clustering. As with any microarray-based approach, transcript coverage is limited relative to RNA sequencing, and isoform-level or splicing changes cannot be resolved.

### 4.2. Probe Annotation and Gene-Level Summarization

Probe sets were mapped to *Drosophila* gene symbols using the annotation package drosophila2.db via annotate::getSYMBOL(). Probes lacking gene annotation were excluded. When multiple probe sets mapped to the same gene, expression values were collapsed to the gene level by median aggregation across probes, providing robust gene-level estimates.

### 4.3. Differential Expression Analysis

Differential expression analysis was performed using the limma package (version 3.66.0.) [53]. Linear models were fitted with lmFit() and moderated using empirical Bayes shrinkage (eBayes). Pairwise contrasts included: *tbph*^Δ23^ vs. *w*^1118^; *tbph*^Δ142^ vs. *w*^1118^; rescue vs. *tbph*^Δ23^; rescue vs. *tbph*^Δ142^. Genes were considered significantly differentially expressed at FDR < 0.05 (Benjamini–Hochberg correction).

### 4.4. Definition of Shared and Rescued Gene Sets

Genes significantly dysregulated in both mutants relative to control and exhibiting the same direction of change were defined as the shared mutant signature (core 2031). Genes from this shared set that were significantly and oppositely regulated in both rescue comparisons were defined as fully rescued genes (core 496). No additional fold change threshold was applied; inclusion was based solely on FDR < 0.05 and concordant direction of regulation across alleles.

### 4.5. Functional Enrichment Analysis

Gene Ontology (GO) over-representation analysis was performed using clusterProfiler [54,55] for Biological Process (BP), Molecular Function (MF), and Cellular Component (CC) categories. Enriched GO terms were ranked by adjusted *p*-value (FDR), and the top terms were displayed as bar plots, where bar length represents gene count, and bar color encodes enrichment significance using a continuous blue-to-red gradient corresponding to −log10(FDR). KEGG pathway enrichment was conducted using enrichKEGG for *Drosophila melanogaster* (dme), with gene symbols converted to Entrez IDs via (https://bioconductor.org/packages/org.Dm.eg.db, version number 3.22.0 and accessed on 1 February 2026). Significance was defined as adjusted *p*-value (BH) < 0.05. Background gene sets consisted of all genes tested in differential expression analyses [56].

### 4.6. Pathway Visualization

Differential expression values were mapped onto the KEGG Toll and Imd signaling pathway (dme04624) using the pathview package (version 1.50.0) [57]. Gene-level log2 fold-changes were visualized using a blue–white–red color scale (blue = downregulated, red = upregulated). Separate pathway diagrams were generated for mutant vs. control and rescue vs. mutant contrasts.

### 4.7. Heatmap Visualization

Heatmaps were generated using pheatmap. Expression values were z-score-scaled per gene prior to visualization. Samples were ordered by genotype. Heatmaps were produced for the top 300 highest expressed genes and shared mutant signature (core 2031) in Supplementary Figure S2 and the *relish* module in Supplementary Figure S4.

### 4.8. Statistical Environment

All analyses were performed in R using Bioconductor packages, including limma, clusterProfiler, org.Dm.eg.db, drosophila2.db, pathview, and pheatmap. Multiple testing correction was performed using the Benjamini–Hochberg method.

### 4.9. Fly Strains and Maintenance

The following fly strains were used in this study: *w*^1118^-*w*;*tbph*^Δ23^/CyO-GFP (BDSC#93599)-*w*;*tbph*^Δ142^/CyO-GFP (BDSC#93600)-*w*;UAS-TBPH/CyO (BDSC#93601)-*w*;;UAS-TBPH^F/L^/TM6b (BDSC#93781)-*w*;*elav*-GAL4/CyO-*w*;;*elav*-GS-GAL4 (BDSC#43642)-*w*;*rel*^E20^ (BDSC#9457)-*w*;;UAS-Dcr2-*w*;;UAS-TBPH-RNAi (VDRC ID38379). Stocks were maintained on standard cornmeal medium at 25 °C under a 12 h light/dark cycle.

### 4.10. Feeding of Adult Flies with RU486

RU486 was diluted to the desired final concentration (5 mM) in 2% sucrose, and the resulting solution was applied to the surface of standard cornmeal medium. Newborn adults were collected and subsequently transferred to vials containing the drug-supplemented medium.

### 4.11. Climbing Assay

One-day-old adult flies (equal numbers of males and females) were collected and transferred to fresh food vials under standard rearing conditions. Locomotor performance was assessed on day 4 using a negative geotaxis assay. Briefly, flies were placed in a 50 mL graduated glass cylinder marked into three 5 cm intervals. After a 30 s acclimation period, flies were gently tapped to the bottom of the cylinder, and the number of individuals reaching the upper section (>10 cm) within 15 s was recorded. Three independent trials were performed per vial, and the mean value was calculated. In total, 20 flies per single vial were tested, and a minimum of 100 flies per genotype were analyzed.

### 4.12. RNA Extraction and cDNA Synthesis

Total RNA was isolated from whole adult heads using TRIzol reagent (Cat. #15596026, Invitrogen (Thermo Fisher Scientific, Waltham, MA, USA)) according to the manufacturer’s instructions. Genomic DNA contamination was removed using the TURBO DNA-free™ Kit (Cat. #AM1907, Thermo Fisher Scientific (Waltham, MA, USA)). First-strand cDNA synthesis was performed using either SuperScript™ VILO™ Master Mix (Thermo Fisher Scientific) or the SuperScript™ III First-Strand Synthesis System (Cat. #18080-051, Invitrogen), according to the manufacturers’ instructions.

### 4.13. Quantitative Real-Time PCR (qRT-PCR)

Quantitative real-time PCR was performed using Platinum™ SYBR™ Green qPCR SuperMix (Cat. #11744-100, Invitrogen) on a CFX96 Real-Time PCR Detection System (Bio-Rad (Hercules, CA, USA)). Relative gene expression levels were calculated using the endogenous reference genes indicated below. The following primers were used (some primer sequences were originally described by [58]):
**TARGET****SPECIES****FORWARD (5′–3′)****REVERSE (5′–3′)****T_MEL_** **_AMPLICON_***relish**D. mel*GGCATCATACACACCGCCAAGAAGGTAGCTGTTTGTGGGACAACTCGC83 °C*syx1a**D. mel*TGTTCACGCAGGGCATCATCGCCGTCTGCACATAGTCCATAG87 °C*rpl11**D. mel*CCATCGGTATCTATGGTCTGGACATCGTATTTCTGCTGGAACCA86 °C*metchnikowin**D. mel*CATCAATCAATTCCCGCCACCGAGAAATGGGTCCCTGGTGACGATGAG82 °C*attacin c**D. mel*CTGCACTGGACTACTCCCACATCACGATCCTGCGACTGCCAAAGATTG83 °C*drosomycin**D. mel*AGTACTTGTTCGCCCTCTTCGCTG CCTTGTATCTTCCGGACAGGCAGT82 °C*rpl32**D. mel*AAGCGGCGACGCACTCTGTTGCCCAGCATACAGGCCCAAG84.5 °C

Specificity was confirmed by melt curve analysis (single peak, no primer dimers), and no-template controls yielded no amplification. Expression levels were normalized to the housekeeping reference gene (*rpl32* [59] or *rpl11)*.

### 4.14. RNA Immunoprecipitation

Flash-frozen *Drosophila* heads from *elav*-GAL4/UAS-TBPH and *elav*-GAL4/+; UAS-TBPH^F/L^/+ genotypes were homogenized in immunoprecipitation buffer (20 mM HEPES, 150 mM NaCl, 0.5 mM EDTA, 10% glycerol, 0.1% Triton X-100, 1 mM DTT) supplemented with protease inhibitors (Roche, Cat. #11836170001 (Basel, Switzerland)) using a Dounce homogenizer as previously described [60]. Lysates were cleared by centrifugation at low speed (5 min) to remove debris. Protein G magnetic beads (Cat. #10003D, Thermo Fisher Scientific) pre-coated with anti-FLAG M2 antibody (Cat. #F3165, Sigma-Aldrich (St. Louis, MO, USA)) were incubated with cleared lysates for 30 min at 4 °C. Beads were washed five times with immunoprecipitation buffer, and RNA was subsequently extracted using TRIzol reagent (Thermo Fisher Scientific, Waltham, MA, USA).

### 4.15. Statistical Analysis

Statistical analyses were performed using GraphPad Prism (version 10.2.3, GraphPad Software, San Diego, CA, USA). Comparisons were conducted using one-way ANOVA followed by Bonferroni’s post hoc test or two-tailed Student’s *t*-test, as appropriate. Data are presented as mean ± standard error of the mean (SEM). Statistical significance was defined as follows: * *p* < 0.05; ** *p* < 0.01; *** *p* < 0.001; **** *p* < 0.0001.

## Data Availability

The microarray data are deposited in GEO, using the GSE330574 accession number.

## Supplementary Materials

The following supporting information can be downloaded at https://www.mdpi.com/article/10.3390/ijms27125359/s1.
