# Supplementary material for: Loss of TDP-43 Drives Innate Immune Activation Through Relish in Drosophila
Source: Int J Mol Sci. 2026 Jun 13;27(12):5359. doi: 10.3390/ijms27125359 (PMC13299317; doi:10.3390/ijms27125359)
Supplement: Supplementary file 1 [file ijms-27-05359-s001.zip › Supplementary Material.pdf]

# Supplementary Materials

# Loss of TDP-43 Drives Innate Immune Activation through Relish in Drosophila

Giulia Romano[1], Raffaella Klima[1], Fabian Feiguin[2].

[1] International Centre for Genetic Engineering and Biotechnology, Padriciano 99, 34149 Trieste, Italy
[2] Neuronal Aging and Neurodegeneration Laboratory, Department of Life and Environmental Sciences, University of Cagliari, 09042 Monserrato, Italy

Correspondence: giulia.romano@icgeb.org or fabian.feiguin@unica.it

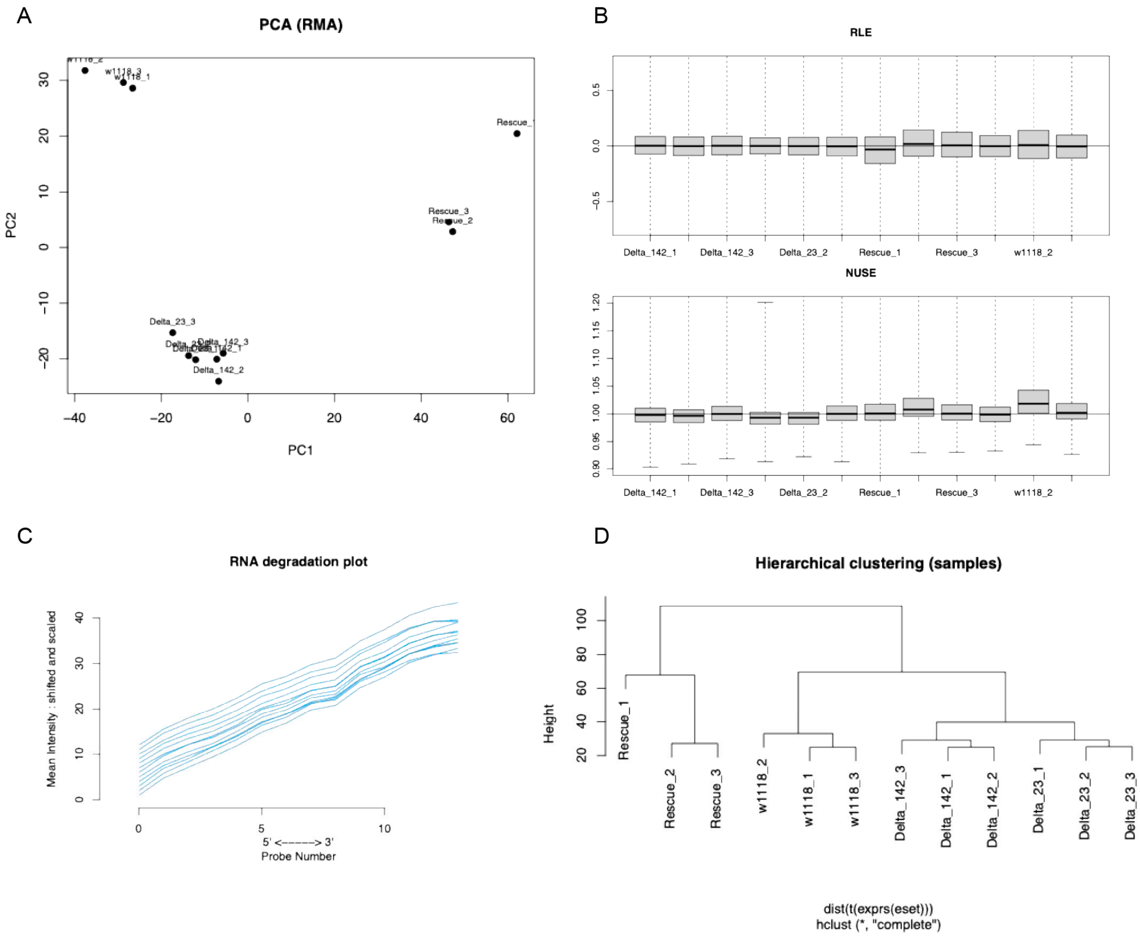


**Supplementary Figure S1. Microarray quality control.** (**A**) Quality assessment of RMA-normalized microarray data. PCA shows clear sample grouping according to genotype. (**B**) RLE and NUSE boxplots display centred distributions with limited variability across arrays, indicating good normalization and technical consistency. (**C**) The RNA degradation plot shows comparable degradation trends among samples. (**D**) Hierarchical clustering (1

– Pearson correlation, complete linkage) further confirms appropriate sample clustering without evident outliers.

A

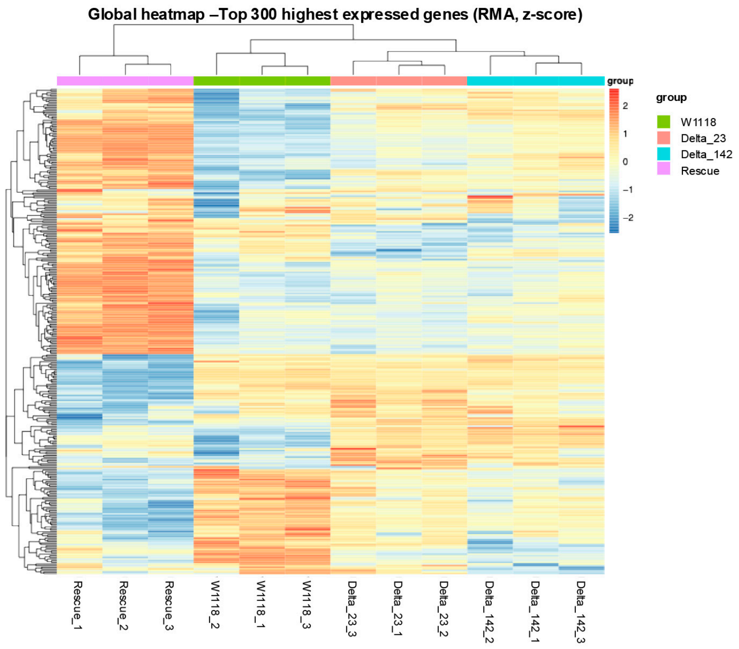


B

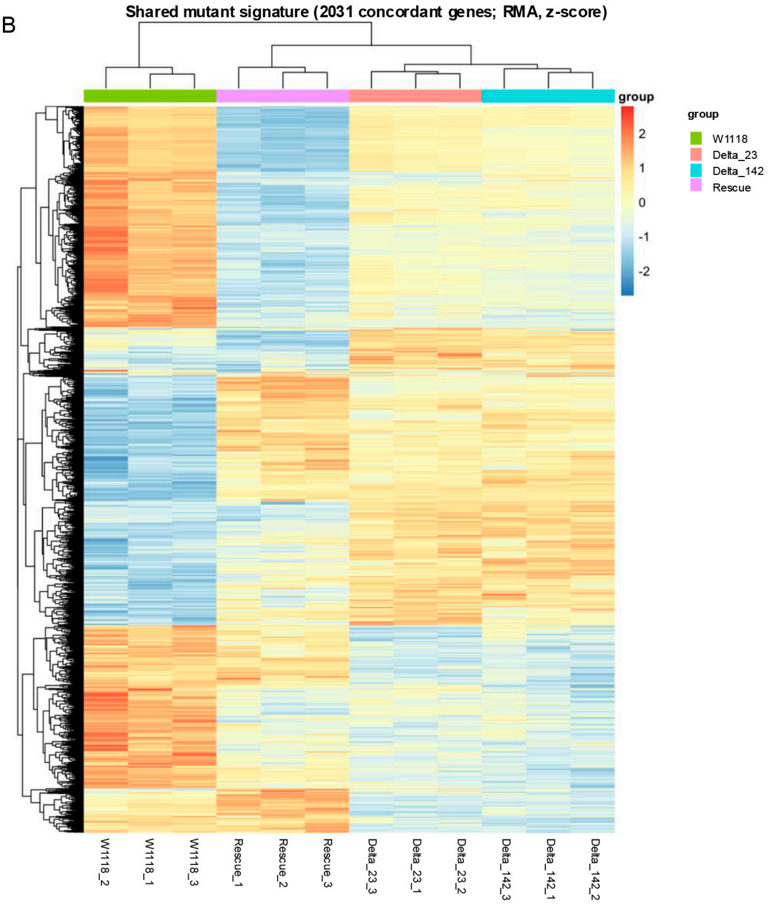

**Supplementary Figure S2. Global and shared mutant transcriptomic signatures.** (**A**) Hierarchical clustering heatmap of the top 300 most highly expressed genes across all samples (RMA-normalized, z-score scaled). Columns represent biological replicates of $w^{1118}$ control (in green), Delta_23 (*tbph*$^{\Delta 23}$) (in pink), Delta_142 (*tbph*$^{\Delta 142}$) (in light blue), and Rescue (*tbph*$^{\Delta 23}$/*tbph*$^{\Delta 23}$,UAS-TBPH;*elav*-GS-GAL4/+) (in violet) groups and rows represent individual genes. Color intensity reflects relative expression levels (red, higher; blue, lower). Unsupervised clustering segregates mutant samples (Δ23 and Δ142) from $w^{1118}$ controls, while Rescue samples cluster closer to controls, indicating restoration of the global transcriptional profile. Replicates within each genotype group cluster tightly, demonstrating high reproducibility. (**B**) Heatmap of 2031 concordantly regulated genes shared between Δ23 and Δ142 mutants (RMA-normalized, z-score scaled). Genes shown exclude discordant transcripts and represent the common mutant core signature. Clustering reveals a consistent and highly overlapping transcriptional pattern between Δ23 and Δ142, characterized by coordinated up- and downregulation relative to control. Rescue samples display a partial reversion toward the $w^{1118}$ expression pattern, supporting functional recovery of the shared mutant transcriptional program.

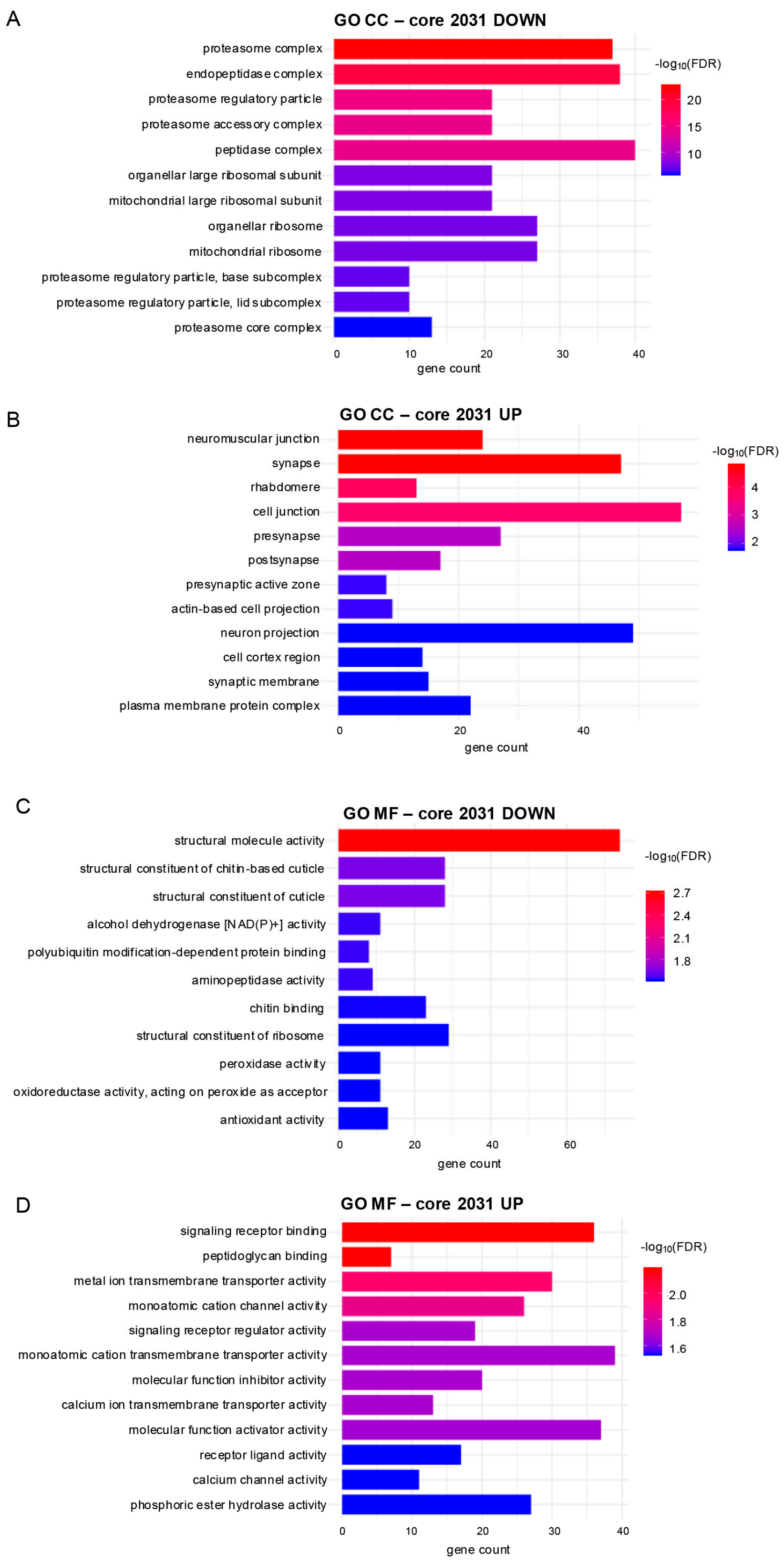


**Supplementary Figure S3. Gene Ontology (GO) Cellular Component and Molecular Function enrichment of the shared mutant transcriptional core.** (**A**) GO Cellular Component (CC) terms enriched among genes commonly downregulated in both *tbph* mutants. Downregulated genes are significantly associated with proteasome-related complexes (proteasome complex, endopeptidase complex, proteasome regulatory particle), ribosomal subunits (organellar and mitochondrial

ribosome), and mitochondrial compartments, consistent with coordinated suppression of protein degradation and ribosomal machinery. (**B**) GO CC terms enriched among commonly upregulated genes. Enriched compartments include neuronal and synaptic structures such as neuron projection, synapse, pre synapse, post synapse, cell junction, and plasma membrane protein complexes, indicating activation of synaptic and membrane-associated programs. (**C**) GO Molecular Function (MF) terms enriched among downregulated genes. Significant categories include structural constituent of ribosome and cytoskeleton, oxidoreductase activity, peptidase activity, and enzyme activities related to metabolic processes, supporting impairment of ribosomal and metabolic functions. (**D**) GO MF terms enriched among upregulated genes. Enriched terms include signaling receptor binding, peptidoglycan binding, ion channel activity (including monoatomic cation channel activity), neurotransmitter receptor activity, and phosphatase regulator activity, consistent with enhanced immune signaling and receptor-mediated processes. In all panels, bar length indicates gene count and color denotes −log10(FDR-adjusted *p* value).

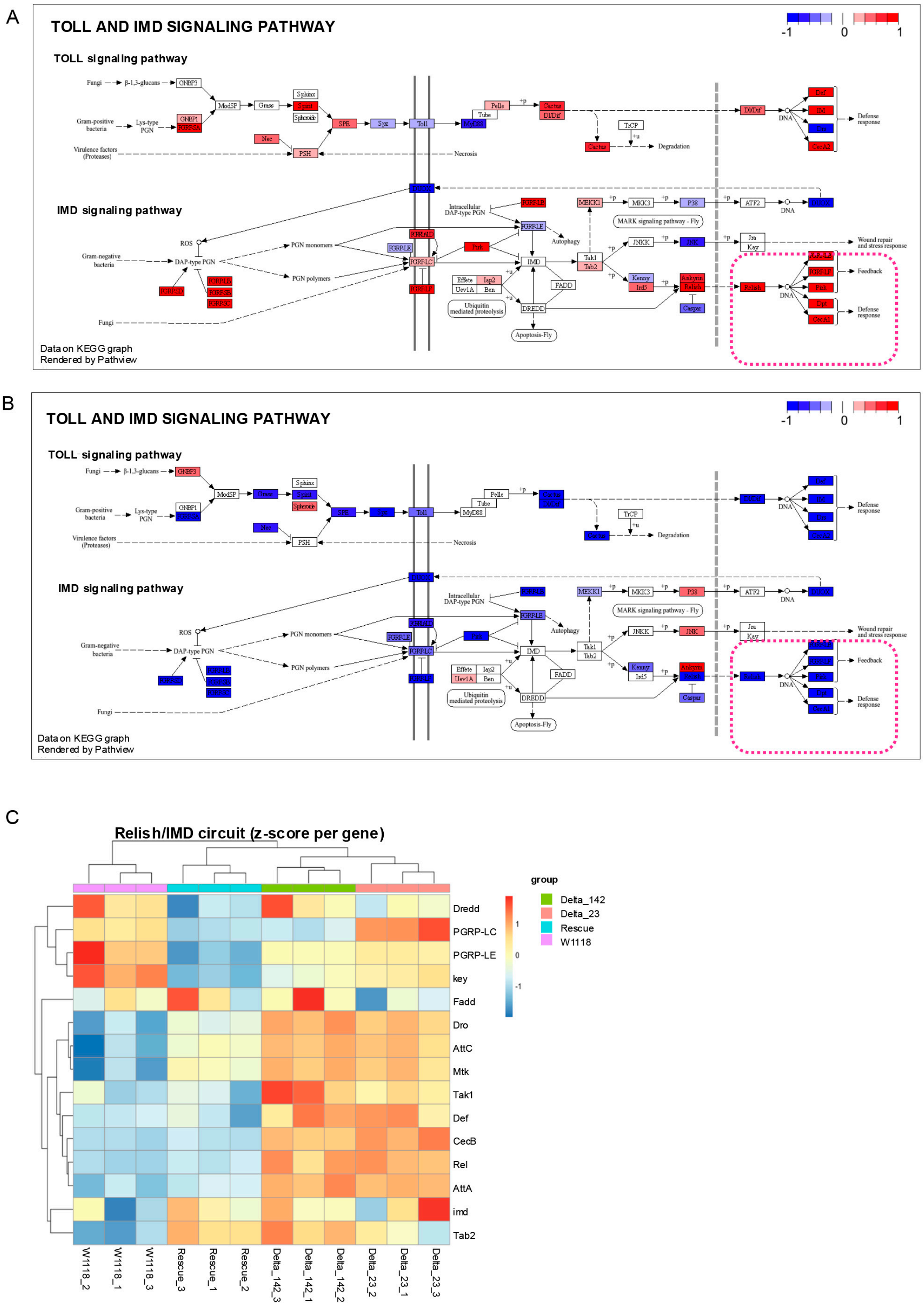


**Supplementary Figure S4. KEGG pathway mapping of Toll and Imd signaling in mutant and rescue conditions.** (**A**) Graphical representation of the KEGG Toll and Imd signaling pathway overlaid with expression changes derived from the shared 2,031-gene mutant core. Nodes are color-coded according to normalized expression changes relative to controls. Coordinated upregulation is observed across multiple pathway components, including upstream receptors, adaptor proteins, intracellular signaling mediators, and downstream NF-κB/Relish-dependent antimicrobial peptide (AMP) effectors (highlighted region). (**B**) Mapping of the 496 rescue-reversed genes onto the same KEGG pathway architecture. Components previously upregulated in mutants show opposite regulation in rescue

conditions, indicating coordinated attenuation of innate immune signaling. The most consistent modulation is observed along the Imd–Relish branch and its downstream AMP targets (highlighted region-pink box). (**C**) Heatmap of genes within the highlighted Imd/Relish–AMP module shown as z-score normalized expression values per gene across genotypes. Hierarchical clustering demonstrates consistent induction in both mutant backgrounds and normalization upon rescue.

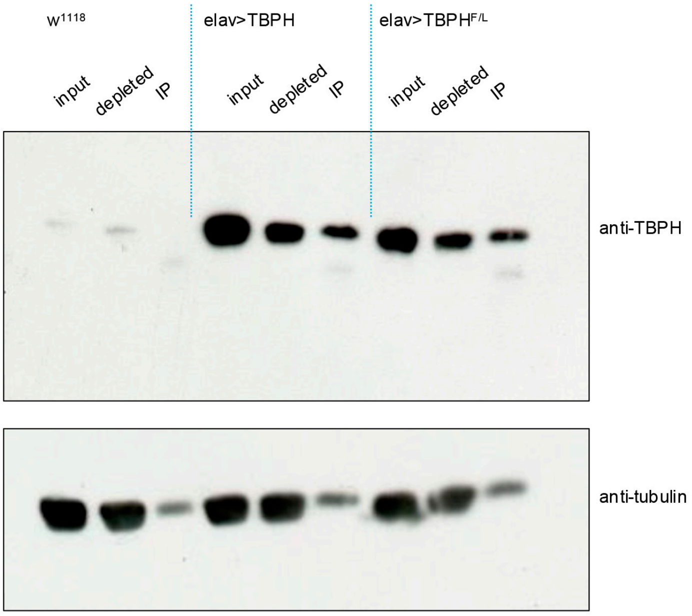


**Supplementary Figure S5. Western blot validation of TBPH and TBPHF/L protein recovery in RIP experiments.** Anti-TBPH immunoblot (upper panel) and anti-tubulin loading control (lower panel) from input, depleted, and immunoprecipitated (IP) fractions of $w^{1118}$ (negative control), elav>TBPH, and elav>TBPH$^{F/L}$ flies. Both wild-type and RNA-binding-deficient TBPH$^{F/L}$ proteins are efficiently recovered in the IP fraction relative to input, with a corresponding reduction visible in the depleted fraction, confirming effective capture of the FLAG-tagged constructs by the beads. Tubulin is used as loading control. In the $w^{1118}$ negative control, endogenous TBPH levels remain unchanged between input and depleted fractions, and no signal is detected in the IP lane.

## Supplementary Materials and Methods

### *Western Blot*

Drosophila adult heads were homogenized in lysis buffer (10 mM Tris, 150 mM NaCl, 5 mM EDTA, 5 mM EGTA, 10% glycerol, 50 mM NaF, 5 mM DTT, 4 M urea, pH 7.4, supplemented with protease inhibitors), and protein content was quantified using the Quant-iT Protein Assay Kit (#Q33211, Thermo Fisher Scientific). Lysates were resolved by SDS-PAGE and transferred to nitrocellulose membranes

(#NBA083C, Whatman) by wet transfer. The following primary antibodies were used: anti-TBPH (1:4000; homemade), and anti-Tubulin (1:2000; #CP06, Calbiochem). Secondary antibodies: anti-mouse-HRP (#31430 Thermo Fisher Scientific 1:10,000) and anti-rabbit-HRP (#31460 Thermo Fisher Scientific 1:10,000).
